# Land use and propolis composition: an analysis of the wax content of *Apis mellifera* propolis

**DOI:** 10.1007/s00442-026-05965-8

**Published:** 2026-09-06

**Authors:** Jenny Michelle Roberts, Philip Donkersley, Stephen Quayle, Allan Edward Watson Rennie

**Affiliations:** 1 School of Engineering, Lancaster University, Lancaster, LA1 4YW UK; 2 Lancaster Environment Centre, Lancaster University, Lancaster, LA1 4YQ UK

**Keywords:** Bee products, Propolis wax, Honeybee, Citizen science, Pollinators

## Abstract

Propolis is a plant-derived resin collected by honeybees, essential for maintaining colony health by protecting against pathogens and building the hive. With the aim of determining influencing factors on the wax content of propolis, 139 samples of propolis were collected from hives across the United Kingdom in 2025 via an extensive nationwide citizen-science approach. We determined propolis mass and wax content, used a generalised linear model to analyse the relationship between colour and wax content, and principal component analysis with generalised linear mixed models to analyse the effect of surrounding land cover on propolis composition. Colour analysis indicated that colour and wax were strongly correlated. Land cover analysis indicated that hives in proximity to larger proportions of arable land and managed grasslands, with greater floral abundance, had increased wax content, compared to hives in proximity to broadleaf woodland, suburban and urban areas with more trees and less grasslands, which tended to have lowered wax content. We conclude that both the colour of raw propolis and land cover surrounding the hive strongly correlates with the wax content of propolis.

**Supplementary Information:**

The online version contains supplementary material available at https://doi.org/10.1007/s00442-026-05965-8.

## Introduction

Honeybee propolis is a resinous material produced when bees collect plant resins and blend them with beeswax to create a protective substance used to seal and maintain the hive. Propolis has been a substance of interest for millennia (Rojczyk et al. 2020), including use as an adhesive (Saccardi et al. 2021), varnish (Stearman et al. 2007), in wound dressings (Andriotis et al. 2020; Da Rosa et al. 2022; Yayehrad et al. 2023) and other pharmacological and cosmetic applications (Crane 2009). The quantity of commercially produced propolis is thought to be in the region of several thousand tonnes per year (Kasote 2017), with the potential to increase this further through government investment in facilities and infrastructure, to support diversification beyond honey production at existing apiary sites, particularly in rural areas of low socio-economic status such as in Indonesia (Harianja et al. 2023). Industries such as medical, pharmaceutical and consumable end products require that propolis harvests are consistent to ensure safety, quality and performance (Katekhaye et al. 2019; Contieri et al. 2022; Sanlier et al. 2026).

ISO24381:2023, the international standard for raw *Apis mellifera* propolis, details the requirements for quality, analysis, packing and other logistics related criteria (International Organization for Standardization 2023). Within ISO24381:2023, the influences of geographical region, plant variation and *A. mellifera* sub species are considered at a global level. More locally, colony health, genetics, hive structure, environment and food availability affect the quality and quantity of propolis produced (Mountford-Mcauley et al. 2021), as well as seasonality and beehive construction material, with post-season and wooden beehives generating the highest yields compared with pre-season and plastic beehives generating propolis with the highest phenolic content and total antioxidant capacity (Kiziltas and Erkan 2021). Foraging behaviour is another contributing factor and bees have been shown to selectively target resin sources that greatly differ in their chemical composition, increasing the diversity of active compounds within the propolis which may enhance protection of the colony against a range of pathological and microbial threats (Drescher et al. 2019).

Wax is a constituent part of propolis, in addition to plant resins, aromatic oils, pollen and other impurities (Burdock 1998; Huang et al. 2014; Anjum et al. 2018). The wax content of propolis is often quoted as 30% - a generalised figure that can be misleading (Salatino and Salatino 2021), with many studies reporting widely varying quantities: 1.5–19.3% (Negri et al. 2000), 13.83–37.58% (Kolaylı et al. 2023), 42–81% (Evran et al. 2023). This may be explained due to variation in geographical region, evidenced by different national standards stating that different values of wax content are acceptable for commercial use (Bankova et al. 2016). For example, the Argentinian standard INA 2004 states that wax content within propolis should not exceed 35% (Stan et al. 2011), the Ukrainian quality standard DSTU 4662:2006 states a maximum of 15%, whilst the Polish standard PN-R-7881 infers that insoluble substances within propolis (including wax) should be below 30% for class 1 and below 50% for class 2, where both classes are considered to be commercial grade (Dvykaliuk 2023). The international propolis standard ISO24381:2023 presents figures of 65% maximum wax content for brown propolis, 30% for green propolis and 60% for red propolis (Annex D ISO24381:2023), highlighting the importance of considering propolis in the context of geographical location. Application governs whether wax within propolis is desirable or not, with healthcare and medical applications often favouring low content (Abdelrazeg et al. 2020), increasing absorption and antimicrobial properties (Rogina-Car et al. 2018), and cosmetic, consumable and food applications more likely to favour high quantities, capitalising on skin moisturising properties (Sahlan et al. 2018) or use as a substitute for palm oil (Fayaz et al. 2017).

We address two key hypotheses: (1) that wax content can be determined by the CIELAB (Standard 2007) colour of raw propolis, with a higher yellowness value (b*) indicating higher wax content and a higher redness value (a*) indicating a lower wax content. (2) that land cover type influences the wax content in propolis, with areas of increased woodland influencing lower wax percentages and areas of greatest floral diversity producing higher wax percentages.

## Materials and methods

Between April and September 2025, beekeepers across the United Kingdom were engaged in a citizen science project to collect propolis from their hives. Each participant was given a propolis collection kit comprising a mesh collection screen measuring 0.46 m^2^, with uniform 2 mm^2^ holes, wooden spacers measuring 0.35 m in length, 0.16 m in width and 0.05 m thick, installation instructions (Online Resource 1) and a collection questionnaire (Online Resource 2). Data collected via the questionnaire included hive location, dates of installation and removal of the collection mesh and bee breed.

### Propolis processing

Each returned propolis mesh was frozen for 2 to 4 h at − 20.3 °C to − 26.4 °C, before being agitated to release the attached propolis. For samples that were extremely sticky, meshes were returned to the freezer a second or third time (< 2 h in total). Processed samples were stored at room temperature (c. 18 ˚C) in a laboratory for further analysis.

### Propolis colour analysis

**Fig. 1:**
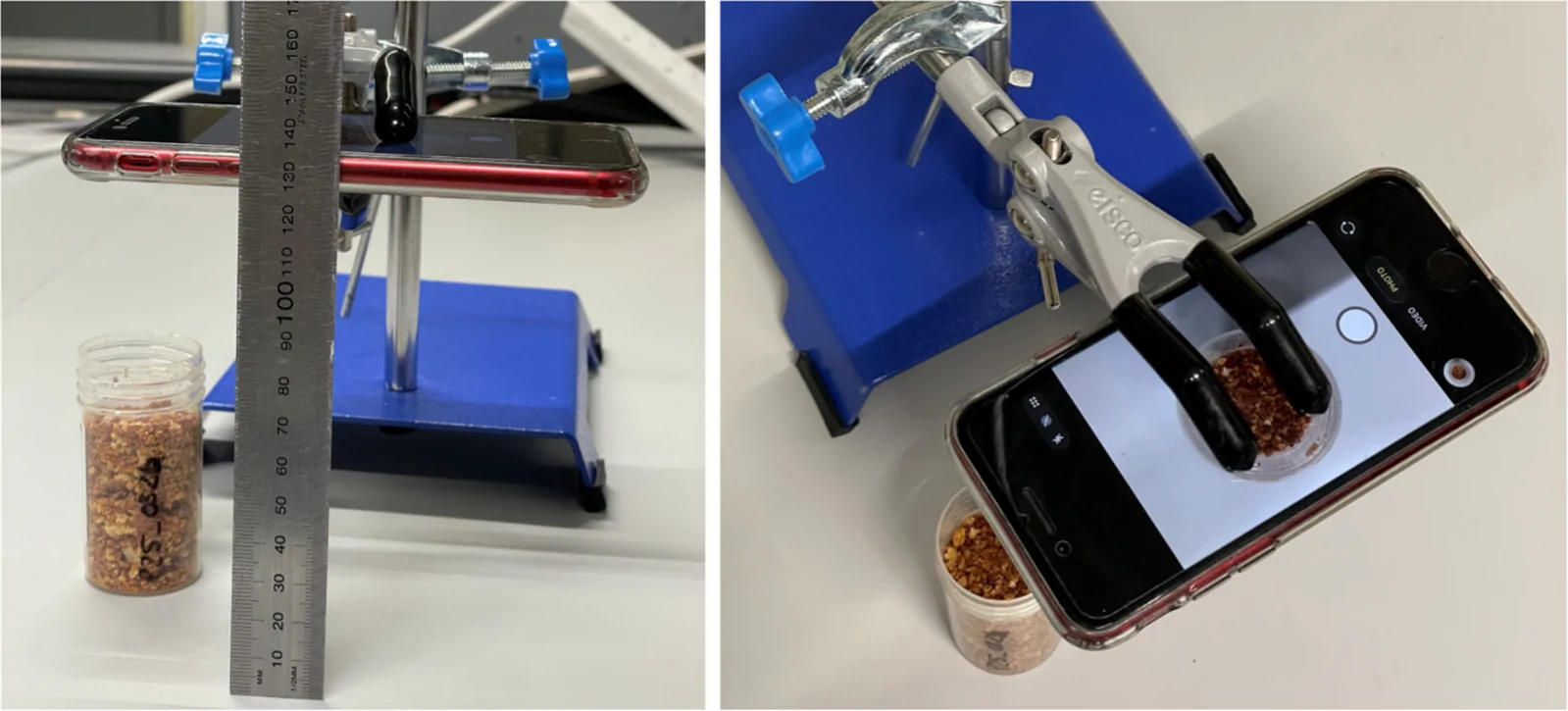
Laboratory setup illustrating camera phone position relative to propolis pot and bench work surface (L) and typical image composition (R)

Digital images of each raw propolis sample were taken using an iPhone SE with 12 MP camera in standard laboratory lighting conditions. Images were taken of each sample pot containing raw propolis at a perpendicular distance of 0.13 m (± 0.01 m) from the laboratory workbench (UK Workbenches, SKU MDL1260/5010) surface (Fig. 1). ImageJ 1.54g (Schneider et al. 2012) was used to develop a batch processing macro to standardise the colour of each image, using the laboratory bench top as a background reference to ensure that the colour differences were dominated by the propolis rather than the lighting conditions:

1. R, G and B values were determined for the background of each image.
2. A neutral target ‘T’ was determined using (R + G+B)/3.
3. Correction factors were determined: R_f_ = T/R, G_f_ =T/G, B_f_ = T/B.
4. Standardised (s) values were determined: Rs = R*R_f_, Gs = G*G_f_, Bs = B*B_f_.

Final Rs, Gs, and Bs values for propolis were converted into L*, a* and b* values, and these data stored in CSV files.

### Wax/resin separation

To preserve both wax and resin elements for physiochemical analysis, and minimise loss of any active ingredients, a separation method similar to Evran et al. (2023), and aligning with that presented in US patent 7,294,351 B2 (Hudnall 2007), was used placing each sealed propolis sample pot in a water bath at 70 ˚C. For each sample, wax was removed from resin via three sequential methods:

1. Liquid wax was poured into an empty wax collection pot. Any wax coating the side of the original pot was scraped off and added to the collection pot.
2. Remnants of wax, towards the end of the process, could be deposited on the relatively cold spatula in layers, by dipping the end repeatedly into the small pool of wax formed on tipping the pot through 45°, being careful not to contact the resin element.
3. In some instances, wax could be scraped gently off the surface of the resin with a spatula, causing a build-up of wax on the end.

Samples were resealed and returned to the water bath and the process repeated for all samples, until as much wax as feasibly possible was removed from the sample.

### Land cover analysis

ArcGIS Pro 3.4.3 was utilised to analyse the land cover within a 5 km and 3 km radius around each hive location from which propolis had been collected. The initial 5 km radius was selected based on research that found honeybee foraging was most efficient at 3 km (Visscher and Seeley 1982; Steffan-Dewenter and Tscharntke 2000), but that they can forage up to 10 km from the hive (Seeley 1986).

The UK Centre for Ecology and Hydrology’s (UKCEH) Land Cover Map 2024 classifies land cover types across the UK to 10 m² pixels as one of 21 categories: broadleaved woodland (BW), coniferous woodland (CW), arable and horticulture (AH), improved grassland (IG), neutral grassland (NG), calcareous grassland (CG), acid grassland (AG), fen marsh and swamp (FMS), heather (HE), heather grassland (HG), bog (BG), inland rock (IR), saltwater (SW), freshwater (FW), supra-littoral rock (SLR), supra-littoral sediment (SLS), littoral rock (LR), littoral sediment (LS), saltmarsh (SM), urban (UB) and suburban (SB).

Area data (m^2^) for each sample was exported and the corresponding percentage land cover for each category calculated as a proportion of the total sample area.

### Data analysis

A Generalised Linear Model (GLM) was fitted in MATLAB (The MathWorks Inc. 2025) using the measured L*a*b* colour values, to determine the relationship between wax content of propolis and colour.

A mixed-effects modelling workflow in R was used to determine the influences on the wax composition of propolis. Data preparation removed samples with missing location data, those that were too small in mass to determine wax content (< 2.9 g, with the exception of one sample of mass 2.29 g whose composition proved suitable for easy separation) and those that used a non-standardised collection method (hive scrapings rather than mesh collection). A log + 1 link function was applied to transform ratio data normal distribution for GLM and handle any zero values in the raw data and a Principal Component Analysis (PCA) carried out on the 21 land cover variables to limit over parameterisation.

A Generalised Linear Mixed Model (GLMM) was fitted using the *lme4* package in R (Bates et al. 2015) and refined via automatic backward model simplification using p-value filtering (*p* ≥ 0.1) with AIC-based stopping criteria using the *lmerTest* package (Kuznetsova et al. 2017). Three iterations were completed to determine the fixed effects of the land cover types and bee breed on the mass of propolis, the mass of wax and on percentage wax by mass. In each model, (1|Sample) was used to control multiple samples from the same apiary, accounting for within and inter-site variance differently. Analysis of the most significant PCs for each model determined land cover composition and was used to infer ecological relationships between landscape structure and the chemical composition of propolis.

## Results

**Fig. 2:**
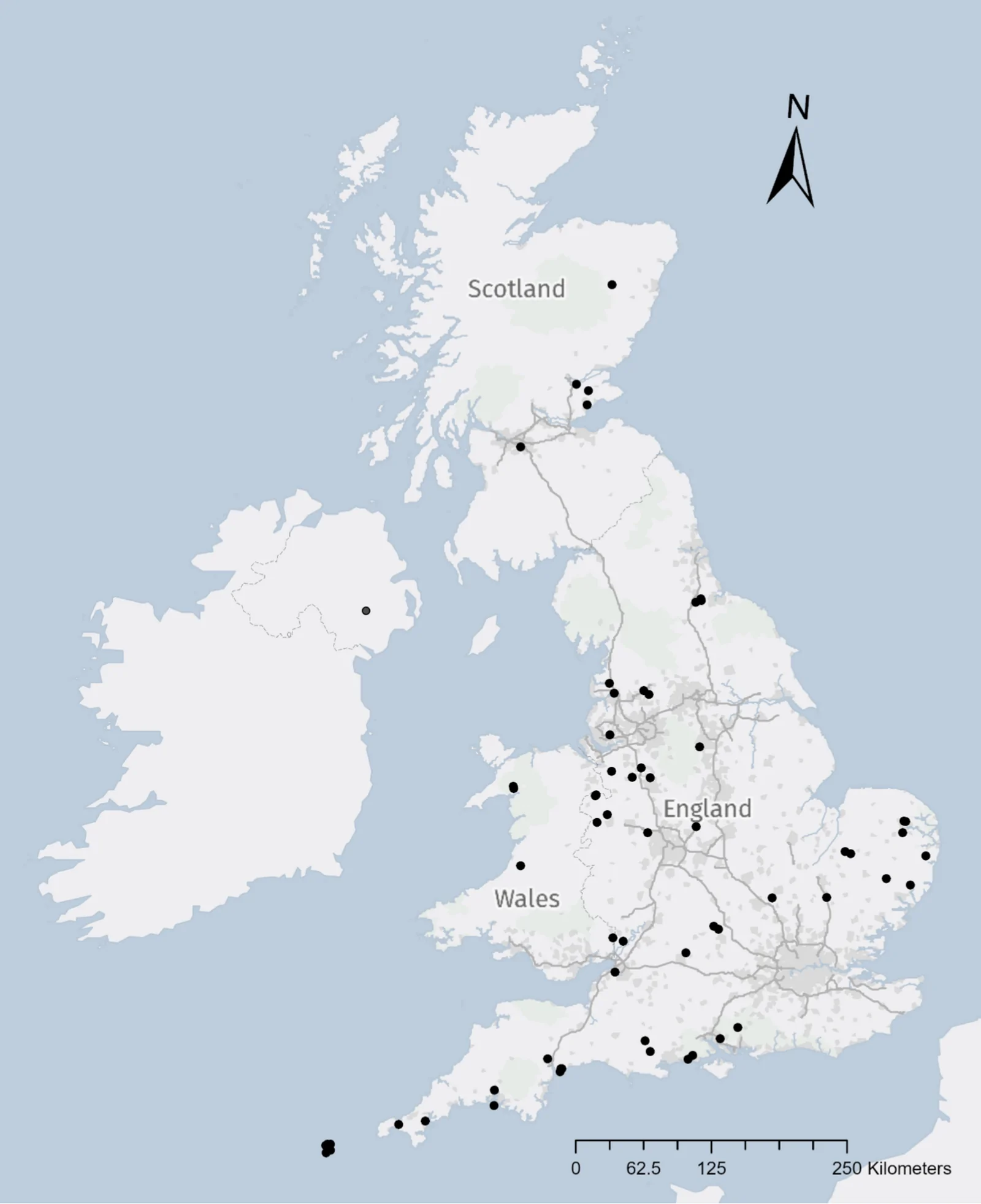
Map of the United Kingdom illustrating the 61 discrete hive locations, denoted by solid black circles, from which the 126 propolis samples were collected from. Contains OS data © Crown copyright and database right 2016. Source: OS Zoomstack (Open Government Licence)

In total, 139 samples of propolis were returned through the citizen science project from around the United Kingdom. Of these 8 were returned without location data and 5 were hive scrapings rather than collected via the provided mesh screen. The remaining 126 samples were deemed suitable for analysis and formed the dataset for this study. These samples were received from 28 different UK counties and 61 distinct locations (Fig. 2), comprising 3 samples from discrete hives at each of 28 apiaries, 2 samples from discrete hives at each of 9 apiaries and 24 discretely located hives. Further refinement of the dataset was undertaken, with 15 samples deemed too small in mass to determine wax content therefore leaving 111 samples suitable for data analysis.

Collection meshes were installed in hives between 28 and 130 days, with duration of installation in the hive showing no correlation with quantity of propolis collected, which ranged from 0 g to 118 g. Bee breed recorded by participants was coded to one of five categories based on questionnaire response: *Apis mellifera mellifera* (AMM, *n* = 87), Buckfast (BUC, *n* = 3), Italian (ITA, *n* = 1), local hybrid (LOC, *n* = 15) and mongrel (MON, *n* = 18). Two responses failed to record any breed data. Wax content ranged from 0% to 77.5% and showed no correlation to quantity of propolis collected.

### Colour analysis

**Fig. 3:**
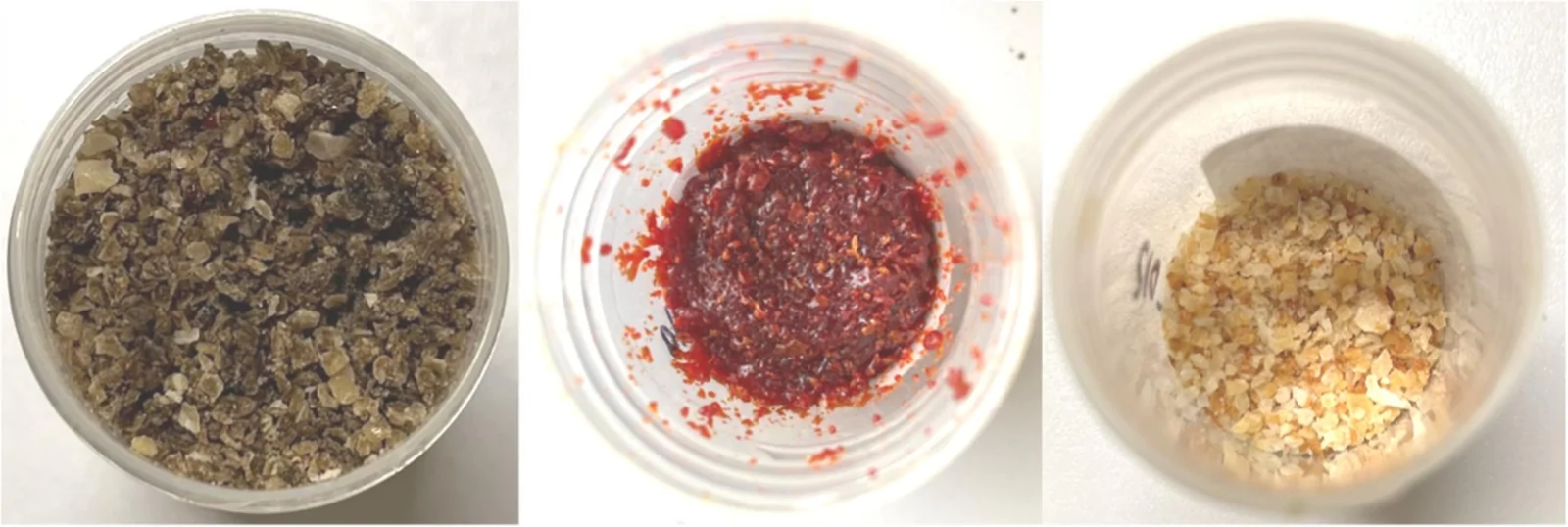
Images illustrating the variation in consistency and colour of propolis received from hives at different apiaries in the United Kingdom: (L-R), grainy brown (hex code #3f2d1c), resinous red (hex code #85312b) and waxy yellow (hex code #bf8323)

Propolis colour and consistency was observed to vary by location (Fig. 3), ranging from brown (hex code #3f2d1c) through to red (hex code #85312b) and yellow (hex code #bf8323), and from being grainy to sticky or waxy, but largely consistent in appearance per apiary.

Colour analysis was undertaken using a tuned GLM model which performed well (F = 46.7, df = 108, *P* < 0.001) and confirmed that higher wax content samples had higher b* values (yellow) (t = 6.506, df = 108, *P* < 0.001) and lower wax content samples had higher a* values (red) (t = −9.366, df = 108, *P* < 0.001).

### Land cover analysis (5 km radius)

**Fig. 4:**
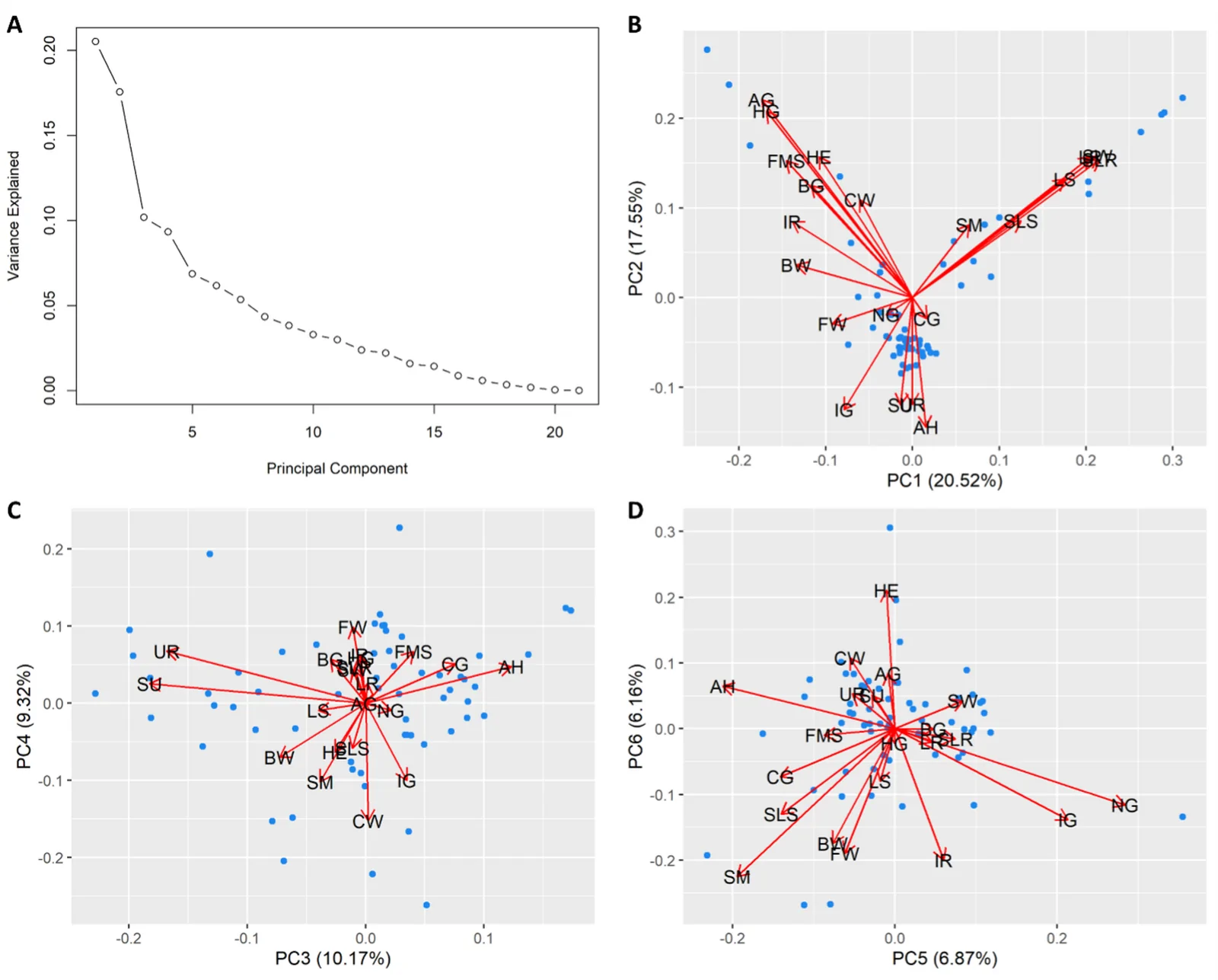
Land cover statistical analysis interrogation at 5 km radius showing (**A**) the PCA scree plot of total variance of each individual principal component (PC), and biplots illustrating key land cover influences and correlations: (**B**) PC1 vs. PC2, (**C**) PC3 vs. PC4, (**D**) PC5 vs. PC6. Biplots were generated in R using the ggplot2 package (Wickham 2016) and the ggfortify package (Tang et al. 2016)

Carrying out a PCA on the 21 land cover variables in a 5 km radius around the hives resulted in principal components 1–6 explaining 70% of the variance and PCs1-13 explaining 95% of variance making it suitable for the GLMM (Fig. 4).

The proportion of wax to propolis was first considered, wherein PC3 (b ± SE = 0.003 ± 0.001, df = 50.43, t = 2.067, *P* = 0.0438) and PC8 were significantly correlated (b ± SE = −0.005 ± 0.002, df = 95.86, t = −2.297, *P* = 0.0238). PC10 (b ± SE = −0.004 ± 0.002, df = 58.62, t = −1.818, *P* = 0.0742) and PC13 (b ± SE = −0.006 ± 0.003, df = 52.30, t = −1.782, *P* = 0.0806) were marginally non-significantly correlated. Interrogating the PC loadings (Table 1) identified that proportion of wax to propolis was negatively correlated with SU, UR and FW land cover types, and positively correlated with AH, NG, FMS and CG. There are also negative correlations with BW and positive correlations from BG, IR and SLS, but to lower significance levels.

**Table 1:** PC loadings for each land cover type at 5 km radius analysis

	PC1	PC2	PC3	PC4	PC5	PC6	PC7	PC8	PC9	PC10	PC11	PC12	PC13	PC14	PC15	PC16	PC17	PC18	PC19	PC20	PC21
BW	−0.227	0.062	−0.235	−0.226	−0.142	−0.322	0.246	0.051	−0.163	−0.429	−0.143	−0.469	−0.080	−0.305	−0.073	0.066	0.044	0.224	0.022	0.111	0.161
CW	−0.103	0.185	0.006	−0.492	−0.103	0.199	0.144	−0.255	0.140	0.294	−0.035	−0.305	0.071	0.203	0.435	0.185	0.118	−0.096	0.032	−0.270	0.099
AH	0.026	−0.245	0.396	0.154	−0.392	0.120	−0.249	0.005	0.107	−0.085	0.273	−0.165	−0.016	−0.042	−0.006	−0.033	0.242	0.089	0.028	−0.011	0.580
IG	−0.134	−0.212	0.112	−0.322	0.393	−0.255	0.141	−0.065	−0.301	0.114	−0.090	0.439	0.044	0.108	−0.035	−0.029	0.163	0.104	0.022	0.005	0.468
NG	−0.052	−0.032	0.068	−0.030	0.523	−0.213	−0.221	0.425	0.510	0.200	−0.028	−0.326	0.108	−0.119	0.004	−0.022	0.034	0.047	0.015	−0.009	0.075
CG	0.029	−0.039	0.244	0.165	−0.260	−0.135	0.575	0.326	−0.033	0.479	−0.162	−0.086	−0.258	0.009	−0.049	−0.201	−0.102	−0.025	0.048	−0.009	0.021
AG	−0.295	0.375	−0.004	−0.002	−0.016	0.155	−0.083	0.008	−0.003	0.045	−0.057	0.020	0.099	0.060	0.205	−0.475	0.008	−0.115	−0.061	0.644	0.148
FMS	−0.247	0.259	0.130	0.217	−0.162	−0.018	0.070	0.341	0.117	−0.153	−0.290	0.265	0.191	0.189	0.065	0.579	0.215	0.004	−0.081	0.063	0.031
HE	−0.183	0.267	−0.084	−0.205	−0.020	0.388	0.164	−0.058	0.350	0.106	0.139	0.243	−0.129	−0.265	−0.527	0.004	0.044	0.273	−0.023	−0.070	0.076
HG	−0.286	0.353	−0.013	0.189	−0.001	−0.041	−0.148	0.141	−0.156	−0.140	−0.065	0.069	0.071	−0.013	0.050	−0.372	−0.120	−0.047	0.187	−0.667	0.138
BG	−0.198	0.212	−0.096	0.185	0.088	−0.001	−0.262	0.018	−0.526	0.485	0.267	−0.231	−0.068	−0.098	−0.162	0.323	0.079	0.045	−0.040	0.060	0.010
IR	−0.235	0.144	−0.016	0.204	0.111	−0.369	0.073	−0.259	0.277	−0.108	0.365	0.073	−0.579	0.269	0.142	0.073	−0.001	−0.018	−0.016	0.018	0.008
SW	0.362	0.268	−0.035	0.142	0.155	0.078	0.071	−0.112	0.022	−0.041	−0.119	−0.065	−0.015	−0.001	−0.016	0.236	−0.610	−0.065	−0.091	0.051	0.514
FW	−0.157	−0.049	−0.035	0.319	−0.114	−0.350	0.079	−0.557	0.227	0.242	−0.151	0.018	0.453	−0.258	−0.089	−0.013	0.027	−0.052	−0.017	−0.016	0.018
SLR	0.366	0.261	−0.031	0.152	0.137	−0.029	0.178	−0.019	−0.064	−0.050	0.055	−0.062	0.051	−0.008	0.080	−0.188	0.433	0.164	−0.652	−0.155	0.025
SLS	0.212	0.146	−0.036	−0.188	−0.259	−0.239	−0.439	0.000	0.059	0.192	−0.344	0.270	−0.377	−0.377	0.225	0.018	0.041	0.094	−0.055	−0.001	0.005
LR	0.348	0.266	0.005	0.083	0.084	−0.037	−0.058	−0.176	0.003	−0.009	−0.288	−0.164	−0.087	0.310	−0.307	−0.058	0.383	0.055	0.539	0.082	0.020
LS	0.298	0.224	−0.128	−0.031	−0.034	−0.147	0.230	0.185	0.009	−0.007	0.506	0.217	0.233	−0.320	0.265	0.067	0.121	−0.098	0.409	0.075	0.038
SM	0.111	0.138	−0.124	−0.329	−0.359	−0.415	−0.172	0.155	0.066	0.087	0.219	0.025	0.244	0.420	−0.348	−0.080	−0.171	−0.038	−0.187	−0.001	0.012
UR	−0.001	−0.202	−0.542	0.218	−0.097	0.098	−0.011	0.084	0.074	0.163	−0.021	0.056	0.071	0.250	0.252	−0.057	−0.061	0.627	0.106	0.011	0.116
SU	−0.024	−0.203	−0.589	0.082	−0.052	0.094	0.017	0.126	0.086	0.050	−0.087	0.024	−0.153	−0.013	−0.118	−0.004	0.249	−0.614	−0.075	−0.055	0.272

Propolis mass was marginally non-significantly correlated with PC2 (b ± SE. = −1.048 ± 0.055, df = 52.46, t = −1.912, *P* = 0.061), indicating a negative correlation with AG and HG. It was also significantly correlated with PC10 (b ± SE. = 0.291 ± 0.118, df = 70.03, t = 2.465, *P* = 0.016), indicating positive correlation with BG and CG and negative correlation with BW.

Wax mass was significantly correlated with PC2 (b ± SE. = −0.097 ± 0.043, df = 54.23, t = −2.231, *P* = 0.029), indicating a negative correlation with AG and HG.

Finally, when considering bee breed, we found that both BUC (b ± SE = −0.032 ± 0.015, df = 31.46, t = −2.061, *P* = 0.048) and MON (b ± SE = −0.013 ± 0.006, df = 47.99, t = −2.002, *P* = 0.050) were significantly correlated with proportion of wax to propolis.

When considering the effects of bee breed, caution must be applied given the low numbers of samples from BUC (*n* = 3), all of which were located at the same apiary. However, greater confidence can be given to the influence of those bees classed as MON (*n* = 16), with samples having a mean wax content of 23% (SD = 19%), compared with the AMM breed category (*n* = 76) whose mean wax content was 30% (SD = 19%).

### Land cover analysis (3 km radius)

The land cover analysis was repeated for areas around each hive with a 3 km radius. The proportion of wax to propolis was first considered, wherein PC5 (b ± SE = 0.004 ± 0.002, df = 47.75, t = 2.399, *P* = 0.020) was significantly correlated and PC10 (b ± SE = −0.005 ± 0.003, df = 40.56, t = −1.810, *P* = 0.078) was marginally non-significantly correlated. Interrogating the PC loadings (Table 2) identified that proportion of wax to propolis was negatively correlated with IG and NG and positively correlated with AH land cover types. There are also negative correlations with BW and positive correlations from NG, FW and SM, but to lower significance levels.

**Table 2:** PC loadings for each land cover type at 3 km radius analysis

	PC1	PC2	PC3	PC4	PC5	PC6	PC7	PC8	PC9	PC10	PC11	PC12	PC13	PC14	PC15	PC16	PC17	PC18	PC19	PC20	PC21
BW	−0.201	0.207	−0.134	−0.157	−0.272	−0.290	0.217	0.061	0.307	−0.320	−0.355	−0.200	−0.208	−0.201	−0.010	0.089	−0.050	0.326	0.247	0.000	−0.204
CW	−0.066	0.205	0.172	−0.514	0.220	−0.177	−0.039	−0.079	0.133	0.076	0.027	−0.100	−0.360	0.047	0.054	0.216	0.211	−0.494	−0.229	0.020	−0.126
AH	−0.027	−0.199	0.323	0.355	0.372	−0.126	−0.175	0.202	−0.108	−0.017	−0.269	0.122	−0.086	−0.049	0.084	0.143	−0.093	−0.006	0.073	0.029	−0.594
IG	−0.157	−0.095	0.323	−0.159	−0.462	0.149	0.121	−0.060	0.043	−0.098	0.524	0.080	0.204	0.078	0.111	0.116	−0.059	0.008	0.032	0.013	−0.461
NG	−0.044	−0.032	0.116	−0.027	−0.355	0.463	−0.171	−0.065	0.185	0.606	−0.415	−0.064	−0.118	−0.100	−0.005	0.031	0.000	−0.001	0.008	0.003	−0.075
CG	0.060	−0.053	0.123	0.285	−0.072	−0.454	−0.105	−0.473	−0.038	0.279	0.215	−0.476	0.024	−0.273	−0.110	0.043	0.015	0.074	−0.061	0.009	−0.019
AG	−0.133	0.366	0.037	−0.229	0.232	−0.026	−0.183	−0.121	−0.183	0.294	0.241	0.214	−0.266	0.142	−0.129	−0.191	−0.232	0.454	0.239	−0.014	−0.087
FMS	−0.159	0.330	−0.098	0.346	−0.061	−0.060	−0.112	−0.090	0.183	0.025	−0.049	−0.193	0.129	0.678	0.087	0.011	0.011	−0.259	0.285	−0.011	−0.049
HE	−0.111	0.251	0.059	−0.274	0.193	−0.014	0.207	−0.249	−0.277	0.093	−0.341	−0.008	0.689	−0.087	0.084	0.054	−0.009	0.005	0.003	0.009	−0.098
HG	−0.165	0.428	−0.191	0.265	−0.077	0.056	−0.097	0.087	0.022	−0.037	0.033	0.066	0.017	−0.031	0.059	0.036	−0.011	0.184	−0.763	0.037	−0.163
BG	−0.156	0.386	−0.176	0.235	−0.042	0.098	−0.066	0.115	−0.095	0.038	0.207	0.201	0.005	−0.566	−0.010	0.091	0.099	−0.385	0.363	−0.011	−0.003
IR	−0.099	0.068	0.063	0.137	0.124	0.159	0.675	0.344	−0.264	0.260	0.115	−0.398	−0.184	0.067	−0.009	0.017	0.004	0.021	−0.006	0.005	−0.011
SW	0.422	0.142	−0.077	0.016	0.088	0.220	0.077	−0.128	0.088	−0.115	0.013	−0.081	0.014	−0.004	−0.320	−0.377	0.510	0.046	0.042	−0.107	−0.411
FW	−0.072	−0.106	−0.069	0.129	0.232	−0.165	0.427	−0.128	0.621	0.308	0.091	0.412	0.098	−0.043	0.013	−0.090	−0.038	−0.011	−0.042	−0.002	−0.018
SLR	0.440	0.142	−0.049	0.059	−0.093	0.020	0.163	−0.195	−0.092	−0.016	−0.042	0.132	−0.147	0.035	0.209	0.111	−0.101	−0.011	0.054	0.765	−0.038
SLS	0.293	0.123	0.005	−0.144	0.186	0.025	−0.232	0.373	0.325	0.112	0.191	−0.332	0.229	−0.145	0.518	−0.122	−0.068	0.139	0.075	0.010	−0.007
LR	0.427	0.156	−0.043	−0.007	0.086	0.157	0.041	−0.005	0.150	−0.045	0.050	−0.054	0.068	0.019	−0.379	0.541	−0.457	−0.024	−0.005	−0.276	−0.029
LS	0.363	0.105	−0.026	0.043	−0.278	−0.253	0.166	−0.070	−0.247	0.116	−0.109	0.244	−0.171	0.051	0.442	−0.087	−0.039	−0.072	−0.022	−0.539	−0.053
SM	0.161	0.051	0.026	−0.108	−0.281	−0.463	−0.075	0.526	−0.064	0.306	−0.021	0.154	0.226	0.114	−0.379	0.027	0.159	−0.019	−0.013	0.166	−0.015
UR	−0.048	−0.248	−0.548	−0.070	0.085	0.019	−0.060	−0.046	−0.104	0.182	0.111	0.026	0.003	0.103	0.169	0.521	0.397	0.247	0.092	−0.036	−0.149
SU	−0.053	−0.232	−0.559	−0.167	−0.030	−0.052	−0.029	0.007	−0.100	0.090	0.010	−0.147	0.005	−0.022	−0.073	−0.331	−0.455	−0.316	−0.062	0.064	−0.359

Propolis mass was significantly correlated with PC2 (b ± SE = −0.149 ± 0.057, df = 46.04, t = −2.633, *P* = 0.012), indicating a positive correlation with HG, BG, AG and FMS. It was also marginally non-significantly correlated with PC7 (b ± SE = −0.199 ± 0.101, df = 53.56, t = −1.965, *P* = 0.055), indicating a lower significance positive correlation with IR and FW.

For wax mass the PC2 relationship weakened to marginal non-significance (b ± SE = −0.085 ± 0.047, df = 52.98, t = −1.784, *P* = 0.080) as did the PC7 relationship (b ± SE = −0.135 ± 0.077, df = 54.69, t = −1.744, *P* = 0.087), indicating lower significance positive correlations.

Finally, when considering bee breed, we found that both BUC (b ± SE = −0.030 ± 0.016, df = 31.36, t = −1.906, *P* = 0.066) and MON (b ± SE = −0.012 ± 0.006, df = 48.79, t = −1.899, *P* = 0.064) were marginally non-significantly correlated with proportion of wax to propolis.

## Discussion

This study took a citizen science approach to capture ecological variability at a scale that would be difficult to achieve through conventional sampling, spanning both north–south and east–west gradients across the British Isles. By leveraging this broad geographic coverage, we have shown that variation in propolis wax content is not random but strongly structured by landscape context.

In addressing hypothesis (1), we confirmed that colour can be used as a predictor of composition. Yellow colouring of raw propolis indicates higher proportions of wax, red indicates lower proportions of wax (and therefore a more resinous propolis) and brown indicates a mid-range proportion. Colour therefore proves to be reliable indicator of wax content within raw propolis and could feasibly be used as a tool for early classification of propolis prior to processing. This may offer a means to improve efficiency in commercial operations, particularly in industries such as medical and pharmaceuticals (Abdelrazeg et al. 2020), allowing early assessment against the requirements of international and national standards governing wax content as an indicator of quality (International Organization for Standardization 2023). It may also be of value in the support of diversification of bee products being produced in low socio-economic rural areas (Harianja et al. 2023), offering a low-cost and accessible process for quality assurance.

Addressing hypothesis (2), land-cover composition emerged as a key correlate of propolis composition, with certain environments promoting or constraining wax production and resin collection. Urban and suburban areas, which typically contain greater tree cover and less natural grasslands (Gill et al. 2008), had a negative effect on wax content in propolis. This is consistent with the expectation that landscapes containing more trees provide greater availability of resin sources, allowing bees to incorporate proportionally more resin and less wax into propolis.

Proximity to broadleaf woodland and neutral grassland was also associated with reduced wax content in samples (although these relationships were associated with principal components that explained less variation than those describing urban and suburban land cover). Nevertheless, the direction of these associations is ecologically plausible, as broadleaf woodlands and semi-natural grasslands support a greater diversity of flowering plants and woody vegetation that can provide resin sources for propolis collection.

Conversely, landscapes characterised by arable land and improved grasslands were associated with higher wax content. Although these habitats may support abundant floral resources for nectar and pollen (Nichols et al. 2022), they generally contain fewer resin-producing trees and shrubs (Haines-Young et al. 2003). This suggests that where suitable resin sources are less available, bees compensate by incorporating proportionally more wax into propolis.

These findings are consistent with previous studies demonstrating that land-cover composition influences honeybee foraging behaviour, with habitats such as broadleaf woodland and species-rich grasslands supporting greater resource collection (Donkersley et al. 2014, 2017). Although these studies focused on pollen rather than resin foraging, they support the broader conclusion that landscape composition strongly shapes the availability and exploitation of floral resources by honeybees.

These land cover types also correlated with increased propolis and wax masses, supported in the analysis by significant land cover PCs, albeit propolis mass included the wax proportion and therefore is likely to be heavily influenced by wax mass. Beeswax is produced by the wax gland complex, comprising epithelial cells, oenocytes and adipocytes (Cassier and Lensky 1995), which develop in young honeybee workers at 12 to 18 days old. The production of beeswax requires an increased consumption of sugar (at a ratio of c.20:1 in central Europe) and pollen to offset the energy requirement to produce wax (Bogdanov 2016). In the context of our study, wax is produced by honeybees solely as an ingredient of propolis that is used to prevent draughts or predator ingress, rather than for the production of combs. Therefore, it can be reasoned that propolis is ‘bolstered’ with greater quantities of wax, in the absence of sufficient resin, to achieve the required purpose. This requires sufficient floral sources to ensure the quantity of honey and pollen available for wax producing workers to consume.

Decades of literature published illustrates that *A. mellifera* foraging distances vary (Visscher and Seeley 1982; Seeley 1986; Steffan-Dewenter and Tscharntke 2000), with some reporting proximal foraging at distances below 1 km from the hive (Waddington et al. 1994) and others documenting long-range foraging behaviour, with 50% of bees traveling more than 6 km (Beekman and Ratnieks 2000). Our analysis carried out at two different radii around the hive locations determined that regardless of compositional differences in land cover within these areas, the influences on wax content remained true. We can deduce that bees produce more resinous propolis from areas dominated by trees and an absence of floral diversity, and waxier propolis from more open land type areas like grasslands and marsh lands. However, the current land cover categories assessed are broad in nature and do not identify specific floral taxa, neither is it certain that the bees will travel to forage uniformly throughout the area that has been analysed. Future research should aim to identify specific flora known to be visited by the colony, perhaps through pollen DNA sampling which is a technique carried out already via initiatives such as the National Honey Monitoring Scheme (Oliver et al. 2021).

Nevertheless, we propose that propolis composition can be strategically influenced through hive placement, as landscapes rich in diverse and abundant forage appear to better support the energetic demands of wax production, whereas more homogeneous or resource-limited land-cover types may restrict it. This insight into the factors affecting hive placement could allow for targeted industry specific propolis production, for example low wax content resinous propolis for medical and pharmaceutical applications (Rogina-Car et al. 2018), where the active ingredients, including phenolic compounds and flavonoids, of the resin are most valued (Sforcin and Bankova 2011), or high wax content propolis for cosmetic or food industry applications, where the material properties of the wax are desirable (Fayaz et al. 2017; Sahlan et al. 2018).

During the citizen science process, beekeepers anecdotally commented that some colonies produce more propolis than others, seemingly regardless of location and surrounding land cover. This is verified by the results from apiaries where two or three hives have been sampled and both quantity and percentage wax content of propolis vary. Propolis is used within the hive for multiple purposes: for insulation, for hive health and to prevent predators (Simone-Finstrom and Spivak 2010), and the quantity and quality of propolis produced is influenced by hive structure, environmental factors such as climate and seasonality, food availability and disease prevalence (Mountford-Mcauley et al. 2021). Increased colony health reduces the energy required to maintain an efficient immune system and consequently reserves energy to perform vital tasks such as foraging (Simone-Finstrom et al. 2017), which may promote increased resin collection and wax production.

The collection of propolis via the citizen science had both successes and limitations. The willingness from the beekeeping community to be involved with the research was encouraging, and without them the quantities of propolis available to analyse would have been severely reduced. However, consistency in collection was not always achieved, with comments received back on the collection questionnaires highlighting some difficulties in placing the mesh correctly in hives that were not of the National design and colonies either not moving up into the top super, where the mesh was placed, or not being of sufficient size to have the spare resource to gather propolis. Despite these limitations, the citizen science approach for propolis sample collection remains valid and is one that could be replicated throughout the world to further research in the field of propolis.

The wax separation process used in this study deviates from that presented in ISO24381:2023, in which petroleum ether is used to remove waxes (ISO, 2023). This decision was based on the avoidance of using toxic chemicals that might compromise the composition of the separated resin and wax fractions of propolis, which were retained for subsequent material analysis. The international standard assesses quality in propolis extract predominantly used in downstream chemical assays rather than physical assays. With uncertainty of how this would impact physical behaviour of the fractions of raw propolis, alternative methods were sought. Using a methodology as detailed in US patent 7,294,351 B2 avoided using any chemical processes that might impact physical behaviour, as well as championing a more sustainable approach to material processing (Hudnall 2007). Whilst the methodology does not guarantee complete removal of all wax traces from the resin component, it gives sufficient indication of the wax content to enable broad conclusions to be drawn. Avoiding the use of small samples of propolis in this process aimed to reduce the uncertainty that could be potentially introduced through this method.

This study commenced with a feasible sample size overall however, given the wide variety of land cover in the UK, further categorisation by breed, days of collection and geographical location, the sample size was reduced heavily when analysing against the specific criteria. Therefore, in all cases where influences have been identified, further studies are required to verify the initial findings by targeting propolis collection from hives in areas of specific land cover types, geographical locations and for consistent periods of time.

## Conclusions

The research set out to determine whether wax content of propolis, produced in *A. mellifera* hives across the British Isles, could be successfully determined based on colour and land cover surrounding the hive. The results concluded that colour is a strong indicator of wax content within raw propolis and has the potential to offer a low-cost field assessment of propolis quality, which may support beekeepers in diversifying into commercial propolis production.

Land cover within a 5 km and 3 km radius of the hive was shown to have influence on wax content in propolis, with land cover that is florally diverse producing higher wax contents, and conversely land cover that had more trees and less grasslands producing less wax within propolis. Local climate conditions, individual colony size and health, seasonality of flora and subsequent seasonality of propolis production are also likely influencers of wax content within propolis. Therefore, future research should set out to record as much of this data as possible and constrain, where possible, dates and duration of propolis sample collection.

Targeting the production of high or low wax content aims to benefit the industries that use propolis in their products, reducing the amount of post-processing required that may have a detrimental effect on the active ingredients that make propolis so valuable. In addition, the use of a non-chemical based wax separation process offers a more sustainable method for material processing and comparison with the method presented in ISO24381:2023 is an important next step.

This research considers propolis produced in the UK only and therefore the findings are restricted to this geographical region. However, the model for collecting and assessing the wax content of propolis could be replicated around the globe, to continue to support knowledge creation in this research area and support tailored propolis production for specific applications.

## Supplementary Information

Supplementary Material 1.

Supplementary Material 2.

## Data Availability

The data that support the findings of this study are openly available in Zenodo at https://doi.org/10.5281/zenodo.20289801.

## Code availability

The code that supports the findings of this study are openly available in Zenodo at https://doi.org/10.5281/zenodo.20289801.
