# Supplementary material for: Land use and propolis composition: an analysis of the wax content of Apis mellifera propolis
Source: Oecologia. 2026 Sep 6;208(10):130. doi: 10.1007/s00442-026-05965-8 (PMC13547180; doi:10.1007/s00442-026-05965-8)
Supplement: Supplementary file 1 [file 442_2026_5965_MOESM1_ESM.pdf]

Land Use and Propolis Composition: An Analysis of the Wax Content of *Apis mellifera* Propolis

Roberts, J.*, Donkersley, D., Quayle, S., and Rennie, A.
*Corresponding author: j.m.roberts@lancaster.ac.uk

Propolis screen installation instructions

Please wait until the weather has improved and it is forecast for a couple of weeks of warm temperatures!

1. Place the Maisemore Apiaries propolis screen on top of the upper most super.
2. Add the 4 ventilation strips (one in the middle of each side, no need to use screws, nails or staples for the assembly – see image below).
3. Place the crown board and hive roof back on, over the ventilation strips.

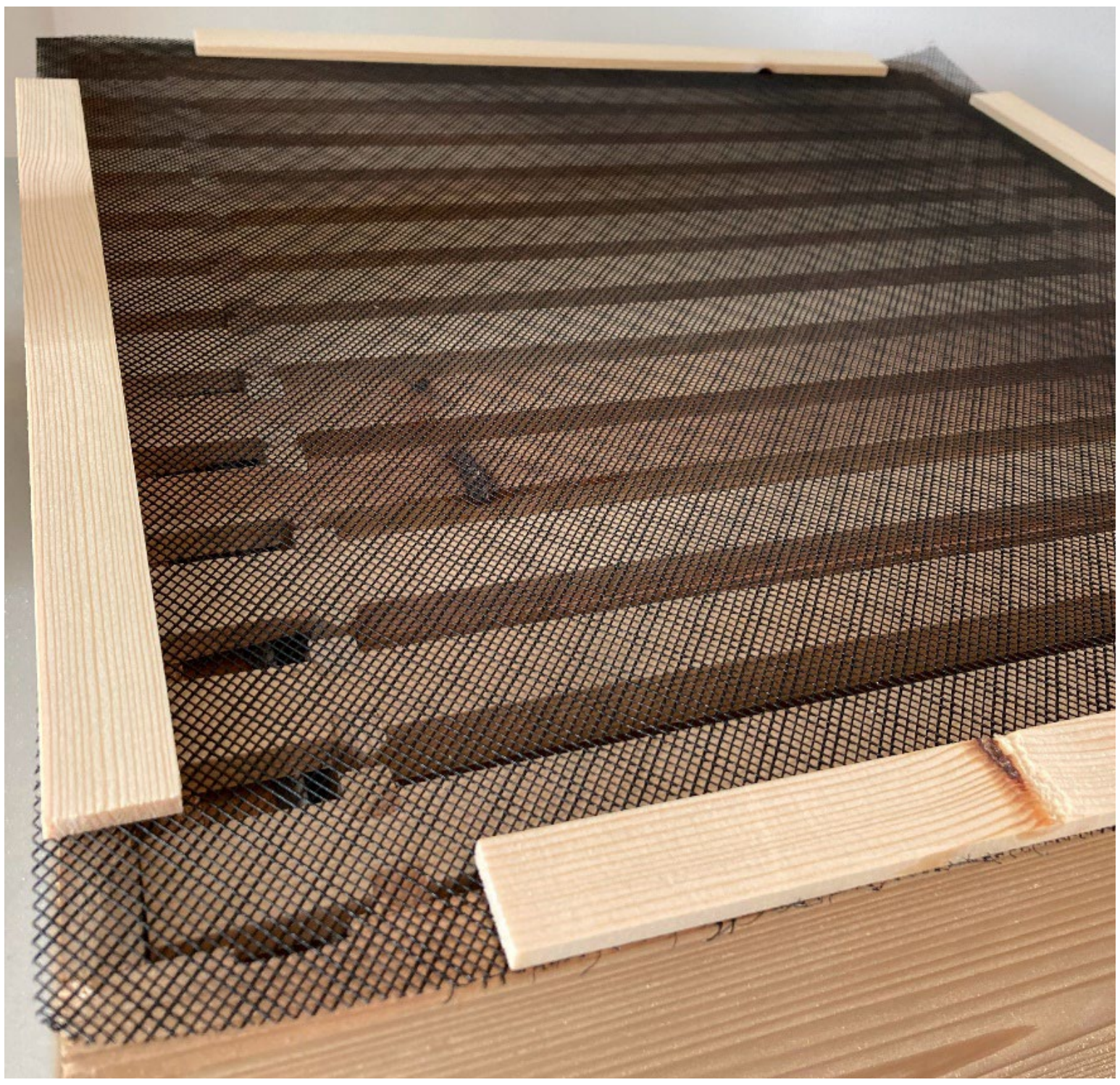

Propolis screen removal instructions

Please leave the propolis screen in place for 2 months (with flexibility depending on weather conditions, your colony requirements and your time).

1. Remove the hive roof, crown board and propolis screen.
2. Replace the crown board and hive roof.
3. Roll the populated propolis screen up and place in the plastic bags provided, securing with rubber bands.
4. Fill in the propolis harvesting information questionnaire.
5. Place the bagged up propolis screen AND the propolis harvesting questionnaire in the postage bag provided (you do not need to return the wooden ventilation strips).
6. If returning more than one sample (from multiple hives in your apiary), please use a different postage bag for each one.
7. Take to your nearest post office to return to Jenny at Lancaster University (postage already paid!).

Many thanks for your support with this project!
