# Supplementary material for: Land use and propolis composition: an analysis of the wax content of Apis mellifera propolis
Source: Oecologia. 2026 Sep 6;208(10):130. doi: 10.1007/s00442-026-05965-8 (PMC13547180; doi:10.1007/s00442-026-05965-8)
Supplement: Supplementary file 2 [file 442_2026_5965_MOESM2_ESM.pdf]

Land Use and Propolis Composition: An Analysis of the Wax Content of *Apis mellifera* Propolis

Roberts, J.*, Donkersley, D., Quayle, S., and Rennie, A.
*Corresponding author: j.m.roberts@lancaster.ac.uk

Propolis harvesting information questionnaire

Thank you for participating in this propolis collection research project! By completing this form and returning the propolis sample you are confirming that:

- You have read the participant information sheet and understand what is expected of you within this study
- You understand that any responses/information you give will remain anonymous
- Your participation is voluntary
- You consent that the data will be pooled and published and that if quotes are provided they could be published
- You consent to Lancaster University keeping the anonymised data for a period of 10 years after the study has finished

Location of hive (please provide a 2 letter, 6 figure grid reference): …………………………………………..

Have you sampled multiple hives at this location for this citizen science project? **Yes/No**

If Yes:

Please note how many hives have been sampled (please circle): 2 3

This is hive (please circle): 1 2 3 of 2 3

Date of installation of propolis screen: ……………………………………………………………………………………

Date of removal of propolis screen*: ……………………………………………………………………………………..

Anticipated date of postage to Lancaster University*: …………………………………………………………………

Colony species: ……

Did you return a propolis sample <u>from this hive</u> for round 1 of the citizen science project? **Yes/No**

Please note any other comments in the space below:

*To allow us to record any variations between time of collection and postage between samples.
